# Psychosocial predictors of mobbing and burnout in para-athletes: Insights for mental health

**DOI:** 10.1371/journal.pone.0331612

**Published:** 2025-09-09

**Authors:** Yeliz Ay Yıldız, Gokhan Buyukluoglu, Nihan Buyukluoglu, Sabriye Ercan, Aydan Orscelik

**Affiliations:** 1 Faculty of Sport Sciences, Alanya Alaaddin Keykubat University, Antalya, Türkiye; 2 Department of Sports Medicine, Health Sciences University Gulhane Medical Faculty, Ankara, Türkiye; 3 Department of Psychiatry, Gölbaşı Şehit Ahmet Özsoy State Hospital, Ankara, Türkiye; 4 Department of Sports Medicine, Süleyman Demirel University Medical Faculty, Isparta, Türkiye; SARAH Network of Rehabilitation Hospitals: Rede SARAH de Hospitais de Reabilitacao, BRAZIL

## Abstract

Para-athletes may experience psychological challenges such as mobbing and burnout, which can impair their performance, motivation, and well-being. Despite the inclusive goals of the Paralympic Movement, recent evidence suggests that para-athletes are not immune to negative psychosocial experiences. This study aimed to examine the relationship between mobbing exposure and burnout among para-athletes and to identify demographic and psychological predictors of mobbing. This cross-sectional study included para-athletes aged 18–45 with at least two years of sports experience. Participants completed an online survey including demographic variables, the Negative Acts Questionnaire-Revised (NAQ-R), and the Athlete Burnout Questionnaire (ABQ). Statistical analyses included group comparisons, correlation tests, and linear regression modeling to explore factors associated with mobbing exposure. A total of 93 para-athletes participated. NAQ-R scores varied significantly by age and showed positive correlations with ABQ-PEE (physical/emotional exhaustion) and ABQ-SD (sport devaluation) scores. Regression Analysis, Adjusted R² = 0.296. Model was significant (F(9,83)=4.10, p < 0.001). Significant predictors, ABQ-SD (β = 0.312, p = 0.004), education level (β = 0.278, p = 0.011). Regression analysis revealed that higher ABQ-SD and educational level were significant predictors of increased mobbing exposure. ABQ-SD and education level are key predictors of mobbing and burnout in para-athletes, revealing that negative interpersonal dynamics persist even in para-sport environments. Targeted anti-mobbing strategies and tailored psychological support are essential to protect mental health and enhance the athletic experience.

## Introduction

Mobbing, also known as workplace bullying, is a prolonged form of psychological abuse involving repeated negative actions aimed at intimidating or isolating an individual. It is a well-documented occupational hazard that transcends industry, gender, and cultural boundaries and has significant consequences for emotional and physical health [1,2]. Although typically studied in professional settings, mobbing is also prevalent in sports environments, where pressure from coaches, teams, and institutions may facilitate harmful interpersonal dynamics [3,4]. In light of these considerations, the assessment of mobbing perceptions within the sporting domain represents a significant research priority [2]. Recently, media reports on mobbing in sport have highlighted the need for more in-depth and rigorous academic research in this field, with the aim of informing the education of coaches, administrators and athletes [2]. To date, there have been studies conducted on the subject of athlete mobbing perception, it appears that the majority of studies focus on the issue of sexual harassment in positions related to sports [3–8]. Moreover, very few studies have been conducted on the subject of athlete mobbing perception with para-athletes [9,10].

In parallel, athlete burnout is a syndrome marked by physical and emotional exhaustion, reduced sense of accomplishment, and sport devaluation. It commonly results from prolonged stress, overtraining, and lack of recovery time, leading to disengagement from the sport and diminished performance [11–14]. Both mobbing and burnout have been associated with deteriorations in self-esteem, motivation, and mental health [15]. Athlete burnout has been shown to exert significant impacts on both mental health and sport performance. A recent systematic review and meta-analysis by Glandorf et al. demonstrated that burnout is consistently associated with adverse mental health outcomes, including depression, anxiety, and sleep disturbances, whereas its effects on physical health remain less conclusive [16]. In a three wave longitudinal trial, Glandorf et al. reported that, at the between-person level, burnout correlated positively with physical symptoms, illness, depression, and reduced life satisfaction, while at the within-person level, increases in sleep disturbances and depressive symptoms predicted higher burnout, and life satisfaction emerged as a protective factor [17]. In terms of performance, Olsson et al. found that higher overall burnout, reduced sense of accomplishment, and sport devaluation were associated with small-to-moderate but significant declines in performance across multiple sporting disciplines [18]. Drawing on interviews with female Paralympic athletes, Alexander et al. identified that supportive and individualized coaching practices fostered positive personal and performance outcomes, whereas ineffective or harmful coaching—particularly those exacerbating issues related to gender and disability—had detrimental psychological effects [19]. Francisco et al. examined burnout prevalence using two distinct measures, reporting rates ranging from 3.4% to 3.8%, suggesting that while relatively low, burnout remains a relevant concern [20]. Bentzen et al., in a 44-week prospective study of elite Paralympic athletes, found weekly prevalence rates of anxiety and depressive symptoms of 15% and 21%, respectively, and identified significant associations between these symptoms and injury occurrence, illness, and reduced sleep duration [21].

While the global sport community increasingly acknowledges the importance of safeguarding athletes, para-athletes occupy a uniquely precarious position within this discourse. The intersection of disability and elite sport creates layered vulnerabilities—where performance demands are compounded by social prejudice, infrastructural dependence, and systemic barriers that restrict autonomy. These factors not only heighten exposure to mobbing but also intensify its psychological consequences, including burnout, disengagement, and diminished well-being [22]. Despite high-level commitments such as the *Safe Sport* framework and international declarations of athlete rights, protective mechanisms for para-athletes are often underdeveloped, inconsistently enforced, or inaccessible [23]. Addressing this dissonance between institutional ideals and lived realities is critical both to advancing equity in sport and to mitigating preventable harm.

Although the Paralympic Movement promotes ideals of inclusion, empowerment, and equality, para-athletes remain exposed to a spectrum of psychosocial stressors that undermine these principles. Beyond facing comparable levels of bullying and harassment to their non-disabled peers, para-athletes contend with unique challenges, including disability-related stigma, dependency on institutional structures for training and competition, and structural barriers limiting career sustainability [24]. Despite the salience of these risks, empirical research focusing specifically on mobbing in para-sport remains scarce. Very few studies have examined how burnout—a multidimensional syndrome encompassing emotional/physical exhaustion, sport devaluation, and reduced sense of accomplishment—interacts with demographic characteristics to shape vulnerability. This knowledge gap reflects a broader tension between policy discourse and practice in safeguarding, where institutional narratives of protection often fail to translate into effective athlete welfare strategies.

The present study aims to address this theoretical and practical gap by investigating the associations between mobbing exposure, burnout, and key demographic factors among para-athletes. The study hypothesized that: Higher burnout levels, particularly sport devaluation, would be associated with greater mobbing exposure; Demographic variables such as age, gender, and education would influence mobbing risk; National team membership would act as either a protective factor or a risk amplifier.

By clarifying these relationships, the study seeks to generate evidence-based insights to inform targeted psychological and organizational interventions, thereby strengthening the protection and mental well-being of para-athletes.

## Materials and methods

### Research design

The study was cross-sectional in design. Health Sciences University Gulhane Training and Research Hospital Ethics Committee’s decision dated 27/ 02/2024 and numbered 2024/67 approved the study.

All participants provided written consent for their personal data to be stored.

### Description of participants

The study included para-athletes aged 18–45 years who had consented to participate in the online survey. A total sample size of 88 athletes were planned to participate in the study, with a medium effect size and 90% power, α-error (margin of error) 0.05. Ninety-six para-athletes participated in the study. The study excluded three para-athletes with less than two years of athletic experience, as the validity and reliability analysis of the Negative Acts Questionnaire-Revised (NAQ-R) was conducted on individuals with a minimum of two years’ tenure in the same position [25]. A total of 93 para-athletes were included in the study.

### Data collection procedure

Following the approval of the Ethics Committee, the study was initiated on 1 April 2024 and concluded on 25 July 2024. Informed consent was obtained at the outset of the online questionnaire, and those who consented proceeded with the survey. The para-athletes completed the questionnaire on a single occasion. Prior to the commencement of the study, contact was made with the coaches of the para-athletes and federations. The study was explained to the coaches, who then forwarded the survey link to the para-athletes. It was estimated that the completion of the questionnaire would require approximately 15 minutes. With the assistance of coaches and para-athletes, data was gathered over a four-month period.

### Description of instruments

The questionnaire comprised a series of demographic variables, including age, gender, educational status, marital status, years as an athlete, branch, and nationality. Additionally, it incorporated two scales: the NAQ-R and the Athlete Burnout Questionnaire (ABQ).

The NAQ-R, developed by Einarsen and Raknes (1997) and later revised by Einarsen and Hoel (2001, 2009), is one of the most widely used instruments for assessing experienced workplace bullying behaviors. It consists of 22 items grouped into three dimensions: personal derogation/bullying, work-related harassment, and physical intimidation, scored on a five-point Likert scale [26–28]. The NAQ-R translated and psychometrically validated for the Turkish population, where the factor structure comprises two sub-dimensions while retaining the same number of items [25–29]. The NAQ-R has also been applied to athletes with disabilities to explore bullying in sports contexts, supporting targeted prevention and intervention strategies [24].

The ABQ was developed by Raedeke and Smith (2001) for the purpose of evaluating the burnout status of athletes [30]. It is used to measure the emotional, physical, and mental exhaustion that athletes experience as a result of intense training, competition, and the pressures of athletic performance. The ABQ helps to understand the signs of burnout, which can be detrimental to an athlete’s well-being and performance. By assessing emotional exhaustion, depersonalization, and reduced personal accomplishment, the ABQ helps identify athletes who may be at risk of burnout, particularly in a para-athlete context where there may be additional stressors. Understanding and addressing burnout is critical for ensuring the long-term health and success of para-athletes, as it can lead to more effective coaching, better mental health support, and a more sustainable athletic career [11,13]. The questionnaire, which has been validated and found to be reliable in a Turkish context, comprises three subscales, comprising 15 questions and 5 items, which assess emotional and physical exhaustion, a decreased sense of achievement, and a devaluation of sport. The questionnaire employs a five-point Likert scale format [31].

### Statistical analysis

The data were analysed using the SPSS v.23 package programme. The Kolmogorov-Smirnov test indicated that the data were not distributed normally. The Mann-Whitney U and Kruskal-Wallis tests were used to compare NAQ-R and ABQ subscale scores across categorical variables such as age, gender, and athlete’s nationality status. In the correlation analysis, the Spearman correlation test was applied to assess the relationship between NAQ-R scores and ABQ subscale scores. Whereas in the regression analysis, the linear regression test was the preferred method. In linear regression analysis which is aimed to identify the factors influencing mobbing exposure among para-athletes, the dependent variable was the NAQ-R score (mobbing exposure), while the independent variables included other descriptive status such as age, gender, education level, years as an athlete, nationality status, marital status and ABQ subscale scores. These variables were selected due to their potential influence on bullying experiences

Since the data were not found to be normally distributed, they were presented as frequency (n), ratio (%) and median (25th percentile to 75th percentile). A p-value of 0.05 or less was considered statistically significant.

## Results

A total of 93 para-athletes (34 female [36.6%], 59 male [63.4%]) were included in the study. The majority (81.7%, n = 76) held national athlete status. Descriptive statistics for demographic variables and questionnaire scores are presented in Table 1.

**Table 1:** Descriptive characteristics of the participants.

Categorical variables	n	%	NAQ-R	ABQ-RSA	ABQ-PEE	ABQ-SD
Age (years) *						
21-22	20	21.5	29.5 (22.5-43)	12 (8.5-14)	10.5 (7-11.75)	9 (5.25-11)
23-28	16	17.2	42.5 (26.5-63)	12 (8.5-15.75)	12.5 (7.5-15)	12 (7.25-14.75)
29-34	16	17.2	25 (22.25-36.5)	10.5 (6.25-13.75)	12.5 (9.25-13)	9 (6-11.5)
35-40	12	12.9	27.5 (22-34.75)	13 (8-14.75)	10.5 (8.25-14.5)	7.5 (6-13.25)
40 and older	29	31.2	24 (22–33)	10 (8–13)	9 (7–12)	7 (6–11)
Gender *						*
Female	34	36.6	25.5 (22-37.75)	11 (7–13)	9.5 (7–13)	7 (5–11)
Male	59	63.4	29 (23-41)	12 (8–14)	11 (9–13)	10 (6–13)
**Education level**						
Primary school	3	3.2	22 (22–23)	13 (10–14)	10 (9–12)	11 (5–12)
High school	30	32.3	24.5 (22-43)	10.5 (8-13.25)	10 (7–13)	6.5 (5.75-12.25)
Undergraduate	55	59.1	29 (22–37)	12 (7–14)	10 (8–13)	9 (7–12)
Master’s degree	5	5.4	44 (27-68)	11 (7.5-14)	12 (10.5-16.5)	9 (6.5-15.5)
**Years as an athlete (year)**						
2-5	12	12.9	31.5 (23.25-43)	11.5 (7.25-14)	11 (7.25-13)	8 (5–12)
6-8	19	20.4	26 (22-40)	12 (10–13)	9 (8–15)	8 (6–11)
9-11	15	16.2	28 (23-41)	13 (8–15)	10 (9–13)	10 (6–14)
12 and older	47	50.5	27 (22-39)	11 (7–14)	10 (8–13)	9 (6–13)
Nationality status *						
National athlete	76	81.7	29 (22-40.75)	11 (7–13)	10 (7.25-13)	8.5 (6–11)
Non-national athlete	17	18.3	24 (22–34)	13 (11.5-16)	12 (9–15)	10 (6.5-14.5)
**Marital status**						
Single	56	60.2	29 (22-43.5)	12 (8–14)	11 (8–13)	9 (6–12)
Married	35	37.6	27 (22–36)	10 (7–13)	10 (7–13)	7 (6–11)
Widowed	2	2.2	22 (22)	13 (9)	12.5 (9)	12.5 (7)

NAQ-R: Negative Acts Questionnaire-Revised; ABQ: Athlete Burnout Questionnaire; RSA: Reduced Sense of Accomplishment;PEE: Physical/Emotional Exhaustion;SD: Sport Devaluation. *: p value is significant at the 0.05 level (2-tailed). Data were presented as frequency (n), rate (%) and median (25.p-75.p).

The sport disciplines of the participants consisted of 20.4% (n = 19) para-table tennis, 18.3% (n = 17) shooting, 18.3% (n = 17) para-athletics, 9.7% (n = 9) wheelchair basketball and other disciplines (amputee football, badminton, arm wrestling, boccia, judo, archery, seated volleyball, powerlifting, taekwondo), respectively.

Median (25^th^ percentile to 75^th^ percentile) scores were as follows: NAQ-R: 27 (22–40), ABQ-RSA: 12 (8–14), ABQ-PEE: 10 (8–13), ABQ-SD: 9 (6–12) points.

**Fig 1:**
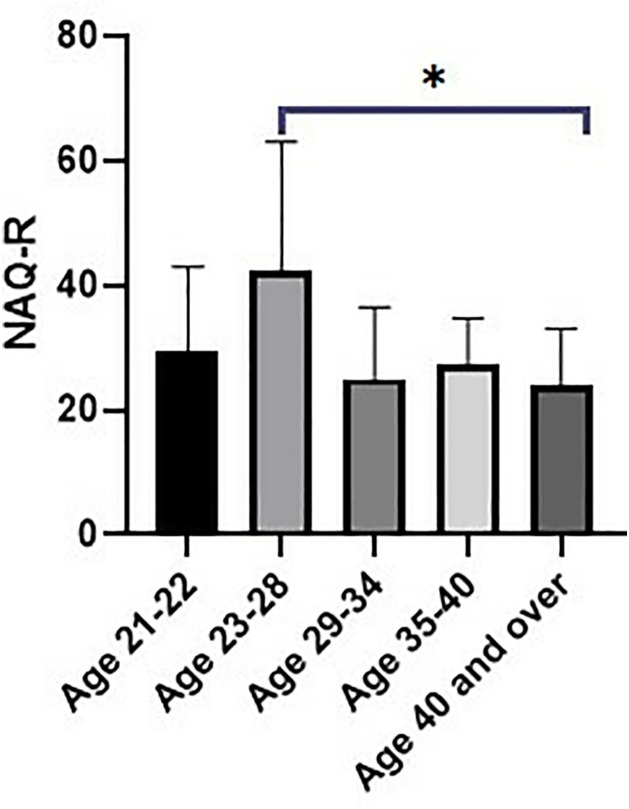
Comparison of NAQ-R scores by age category. Gender: ABQ-SD scores were significantly higher in female athletes (p = 0.023) (Fig 2).

Group Comparisons: NAQ-R scores significantly differed across age groups (p = 0.044), with athletes aged 23–28 and ≥40 showing the highest and lowest mobbing scores, respectively (Fig 1).

**Fig 2:**
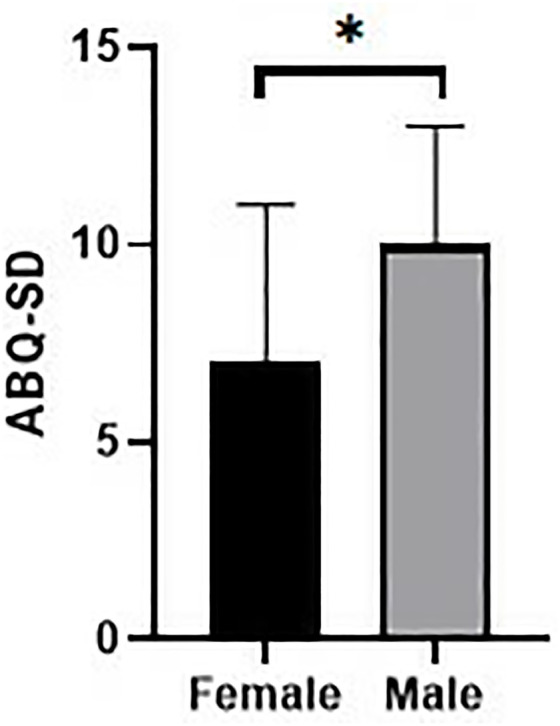
Comparison of ABQ-SD scores according to gender. National Team Status: ABQ-RSA scores were significantly higher in non-national athletes (p = 0.002) (Fig 3).

**Fig 3:**
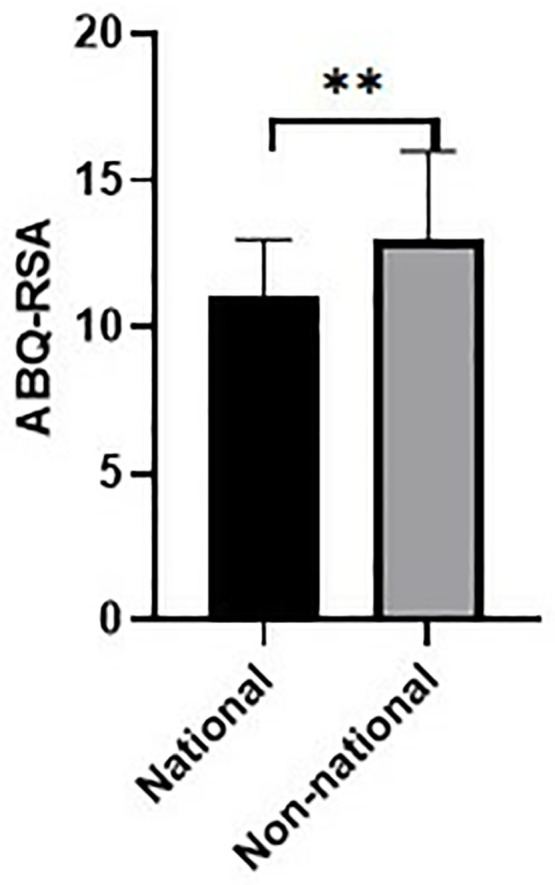
Comparison of ABQ-RSA scores according to national athlete status. NAQ-R was positively correlated with ABQ-PEE (r = 0.231, p = 0.026) and ABQ-SD (r = 0.284, p = 0.006). ABQ-RSA showed moderate positive correlations with ABQ-PEE (r = 0.481) and ABQ-SD (r = 0.594), all p < 0.001. Adjusted R² = 0.296. Model was significant (F(9,83) = 4.10, p < 0.001). Significant predictors: ABQ-SD (β = 0.312, p = 0.004), Education level (β = 0.278, p = 0.011).

## Discussion

This study explored the association between mobbing exposure and athlete burnout among para-athletes, contributing to the limited but growing literature on psychosocial stressors in disability sport. The findings demonstrated a significant positive correlation between mobbing, as measured by the NAQ-R, and burnout dimensions, particularly sport devaluation (ABQ-SD) and physical/emotional exhaustion (ABQ-PEE). ABQ-SD emerged as a central variable, influencing multiple outcomes, including NAQ-R, ABQ-RSA, and ABQ-PEE scores.

### Mobbing and burnout: Theoretical and bidirectional relationships

The observed associations align with chronic stress and emotional exhaustion models, which posit that prolonged exposure to interpersonal mistreatment erodes emotional resources, thereby increasing susceptibility to burnout [32,33]. Conversely, the possibility of reverse causality cannot be excluded: athletes already experiencing burnout—especially high levels of sport devaluation—may be more sensitive to, or more likely to perceive, mobbing behaviors [32]. This bidirectional perspective is supported by theoretical frameworks such as Conservation of Resources Theory and the Job Demands–Resources model, both of which suggest that burnout and mobbing can be mutually reinforcing over time [34,35]. Nevertheless, the cross-sectional design of this study prevents causal inferences, underscoring the need for longitudinal research to clarify the temporal dynamics of these relationships.

### Demographic and institutional variables

In terms of prevalence, the NAQ-R median score among para-athletes in this study was 27, consistent with findings from Buyukluoglu et al., who reported NAQ-R scores of 27 in para-athletes and 26 in non-disabled athletes, with no significant difference between the two groups [24]. Although derived from prior study by the same group, this comparison challenges the assumption that para-sport inherently provides safer or more supportive environments, suggesting instead systemic issues in sport culture regardless of disability status [36,37].

Age-related differences in mobbing exposure were also observed. Athletes aged 23–28 and those ≥40 years reported higher NAQ-R scores than other age groups. This may reflect transitional stressors unique to early-career athletes navigating new professional roles, or to older athletes facing increased physical expectations and decreased social support [24,38]. Gender differences were apparent in burnout dimensions but not in mobbing scores. While NAQ-R did not vary significantly by gender, ABQ-SD scores were higher among female athletes, indicating a greater sense of sport devaluation. This is in line with prior studies suggesting that female athletes may face additional challenges, including gender-based discrimination, lack of institutional support, and persistent sexist attitudes in sport environments [39–41]. However, it is also plausible that male athletes experience sport devaluation differently, potentially due to differing cultural norms regarding emotional expression and vulnerability [40].

Education level was another significant predictor of mobbing. Athletes with higher education levels, particularly those with postgraduate degrees, reported higher mobbing scores. One possible explanation is that more educated individuals possess greater awareness and sensitivity to interpersonal mistreatment and are therefore more likely to recognize and report such experiences [42]. Alternative explanations include heightened expectations for organizational justice and greater likelihood of challenging authority, which may increase exposure to conflict [43].

Institutional affiliation also influenced burnout: non-national athletes reported higher ABQ-RSA and ABQ-PEE scores, suggesting that lack of structured support systems, coaching continuity, and recognition may exacerbate feelings of reduced accomplishment and emotional fatigue [44].

### Practical implications

These findings highlight the need for targeted, intersectional interventions. Anti-mobbing strategies should be integrated into para-sport culture through coaching education, institutional accountability, and athlete advocacy [45,46]. Gender-sensitive programming may help address higher sport devaluation among female athletes, while age- and education-specific approaches could bolster resilience among vulnerable groups [47,48]. Psychosocial support interventions should also incorporate emotional regulation training, as prior research indicates its relevance for Paralympic athletes’ performance and well-being [49]. Crisis-specific strategies are particularly relevant, given evidence from the COVID-19 pandemic that disrupted competition schedules can increase psychological distress [50].

### Limitations and future directions

Several limitations should be considered. The cross-sectional design precludes causal conclusions regarding mobbing and burnout relationships [32,33]. Certain sport modalities, such as judo and boccia, were underrepresented, limiting generalizability across all para-sport disciplines. Self-report measures may introduce social desirability and recall biases, and the sample’s national context limits applicability to international populations. Future research should employ longitudinal or mixed-method approaches, ensure balanced sport representation, and examine additional contextual factors such as team climate, leadership style, and personality traits, which may mediate or moderate these relationships.

## Conclusions

This study identifies sport devaluation (ABQ-SD) and education level as key psychosocial predictors of mobbing and burnout among para-athletes. Findings challenge the assumption that para-sport environments are inherently supportive, revealing that athletes are susceptible to negative interpersonal dynamics and systemic stressors. The results highlight the critical need for targeted anti-mobbing strategies, tailored psychological support, and inclusive, gender-sensitive practices to safeguard mental health and enhance the overall athletic experience in para-sport contexts.
